# Microsurgical resection of fronto-temporo-insular gliomas in the non-dominant hemisphere, under general anesthesia using adjunct intraoperative MRI and no cortical and subcortical mapping: a series of 20 consecutive patients

**DOI:** 10.1038/s41598-021-86165-7

**Published:** 2021-03-26

**Authors:** Henri-Arthur Leroy, Ondine Strachowksi, Constantin Tuleasca, Quentin Vannod-Michel, Emilie Le Rhun, Benoit Derre, Jean-Paul Lejeune, Nicolas Reyns

**Affiliations:** 1 Department of Neurosurgery and Neuro-Oncology, CHU Lille, 59000 Lille, France; 2 Inserm, CHU Lille, U1189-ONCO-THAI-Image Assisted Laser Therapy for Oncology, Univ. Lille, 59000 Lille, France; 3 Department of Clinical Neurosciences, Neurosurgery Service and Gamma Knife Center, Lausanne University Hospital (CHUV) and University of Lausanne (Unil), Faculty of Biology and Medicine (FBM), Lausanne, Switzerland; 4 Department of Radiology, CHU Lille, 59000 Lille, France; 5 Department of Neurosurgery, Lille University Hospital, Rue Emile Laine, 59037 Lille Cedex, France

**Keywords:** Cancer therapy, Head and neck cancer, Neurological disorders

## Abstract

Fronto-temporo-insular (FTI) gliomas continue to represent a surgical challenge despite numerous technical advances. Some authors advocate for surgery in awake condition even for non-dominant hemisphere FTI, due to risk of sociocognitive impairment. Here, we report outcomes in a series of patients operated using intraoperative magnetic resonance imaging (IoMRI) guided surgery under general anesthesia, using no cortical or subcortical mapping. We evaluated the extent of resection, functional and neuropsychological outcomes after IoMRI guided surgery under general anesthesia of FTI gliomas located in the non-dominant hemisphere. Twenty patients underwent FTI glioma resection using IoMRI in asleep condition. Seventeen tumors were de novo, three were recurrences. Tumor WHO grades were II:12, III:4, IV:4. Patients were evaluated before and after microsurgical resection, clinically, neuropsychologically (i.e., social cognition) and by volumetric MR measures (T1G+ for enhancing tumors, FLAIR for non-enhancing). Fourteen (70%) patients benefited from a second IoMRI. The median age was 33.5 years (range 24–56). Seizure was the inaugural symptom in 71% of patients. The median preoperative volume was 64.5 cm^3^ (min 9.9, max 211). Fourteen (70%) patients underwent two IoMRI. The final median EOR was 92% (range 69–100). The median postoperative residual tumor volume (RTV) was 4.3 cm^3^ (range 0–38.2). A vast majority of residual tumors were located in the posterior part of the insula. Early postoperative clinical events (during hospital stay) were three transient left hemiparesis (which lasted less than 48 h) and one prolonged left brachio-facial hemiparesis. Sixty percent of patients were free of any symptom at discharge. The median Karnofsky Performance Score was of 90 both at discharge and at 3 months. No significant neuropsychological impairment was reported, excepting empathy distinction in less than 40% of patients. After surgery, 45% of patients could go back to work. In our experience and using IoMRI as an adjunct, microsurgical resection of non-dominant FTI gliomas under general anesthesia is safe. Final median EOR was 92%, with a vast majority of residual tumors located in the posterior insular part. Patients experienced minor neurological and neuropsychological morbidity. Moreover, neuropsychological evaluation reported a high preservation of sociocognitive abilities. Solely empathy seemed to be impaired in some patients.

## Introduction

Fronto-temporo-insular (FTI) gliomas represent a specific tumor entity, due to their particular evolution and their topography. Previously, FTI gliomas often benefited from a conservative management despite the evolutive nature of these glial lesions^1^. Most of them are low-grade gliomas (LGGs)^2^. Actual guidelines concerning LGGs management confirmed the role of surgery as first line therapeutic option^3,4^. Gross total resection is considered to improve seizures control^5^, prolongs progression-free survival (PFS), overall survival (OS) and delays the malignant transformation^3,4^.

The insular part of these often-large tumors is rarely resected, because of the high risk of strokes and functional impairment. Thus, FTI gliomas were initially biopsied with further complementary radiotherapy and/or chemotherapy^6^. Other series reported a fronto-opercular resection or temporal lobectomy, without significantly reducing the main tumor volume located at the level of the insula. Recently, awake surgery with intraoperative stimulation has emerged as a standard of care for various locations of glioma to increase the extent of resection^7–9^. In case of FTI glioma located in the dominant hemisphere (left-sided in a very majority), we usually perform in our center cortico-subcortical electrical stimulation, especially to avoid eloquent areas, such as speech network. Yet awake surgery does not fit every patient, in particular those presenting with high degree of anxiety, previous neurocognitive impairment with difficulty to keep concentrated, or giant tumors.

The use of intraoperative MRI (IoMRI) has been reported as increasing significantly the EOR of LGGs and high-grade gliomas (HGGs) while preserving function^4,10–12^. The duality between aggressive tumor removal and preserving neural functions is the key in neurological oncology. Applied to the microsurgery of the insular region, IoMRI takes into account the early and important brain shift due to the cerebrospinal fluid depletion secondary to the opening of the Sylvian fissure, and/or the collapse of the operculum, helping surgeon to further readapt his strategy in real intraoperative time.

During last decade, the importance of sociocognitive functions located in the non-dominant hemisphere has been reported, such as mentalizing or emotion recognition^13,14^. Hence, some authors advocate for performing cortico-subcortical stimulation in the case of right fronto-insular glioma to help preserve sociocognitive abilities^15^. However, testing such subtle cognitive functions intraoperatively is demanding and can be long-lasting, especially in case of large FTI glioma (Giant glioma according to Berger–Sanai classification^16^). As a matter of fact, there might be a role of asleep resection of non-dominant FTI gliomas with accurate intraoperative guidance. In our center, we performed such resection under general anesthesia, using the adjunct of IoMRI guidance. Here, we report our results in a series of 20 consecutive patients. The goal of this study was to evaluate the sociocognitive impact of FTI glioma resection located in the non-dominant hemisphere performed under general anesthesia with the help of IoMRI guidance.

## Results

### Demographic data

Twenty patients were included (12 men, 8 women) (Table 1). The median age at diagnosis was 33.5-year-old (range 24–56). Seventeen patients were right-handed, three were left-handed. All patients had a preoperative KPS ≥ 90. Presenting symptoms were as follows: 14 (71%) seizures, 2 (12%) headaches, 3 (15%) incidental findings, 1 (6%) sensitive symptoms (permanent paresthesia of the contralateral hemi-body). In 80% of patients, the preoperative neuropsychological evaluation was normal.

**Table 1:** Descriptive data of the 20 operated patients.

Age at diagnosis	Gender	Preoperative KPS (%)	Berger Sanai classification	Preoperative volume (cm^3^)	Postoperative volume (cm^3^)	Extent of resection (%)	Tumor type	WHO grade	Postoperative treatment	Early postoperative adverse event	KPS at discharge	KPS at 3 months	Neuropsychological evolution at 3 months
27	F	100	1	9.9	0	100	Oligodendroglioma	2	MRI follow-up	–	100	100	Stable
46	M	90	4	41.4	4.6	89	Glioblastoma	4	Radio-chemotherapy	–	90	100	Stable
24	F	90	4	41.5	3.0	93	Anaplastic astrocytoma	3	Radio-chemotherapy	–	90	70	Stable
40	F	90	1 + 4	42.8	4.0	91	Astrocytoma	2	MRI follow-up	–	90	90	Stable
29	M	90	1 + 2	113.9	6.3	94	Oligoastrocytoma	2	MRI follow-up	–	90	100	Stable
44	F	90	2 + 3	80.1	6.1	92	Oligoastrocytoma	2	MRI follow-up	–	90	90	Improvement
48	M	100	1	33.5	3.7	89	Oligodendroglioma	2	Chemotherapy	–	80	90	Stable
33	M	90	4	47.7	1.3	97	Oligoastrocytoma	2	MRI follow-up	Deshinibition	90	90	Stable
32	F	90	1 + 4	98.1	0	100	Astrocytoma	2	MRI follow-up	Apragmatism psychomotor slowdown	80	100	Stable
29	M	90	1	69.0	1.0	99	Astrocytoma	3	MRI follow-up	Diabetes insipidus	80	100	Stable
34	F	100	1	60.0	0	100	Anaplastic oligoastrocytoma	3	Radiotherapy	Left hemiparesis, awakening delay	50	90	Decrease
29	F	100	1 + 4	28.2	0.3	99	Oligodendroglioma	2	MRI follow-up	Transient hemiplegia	80	90	Stable
41	M	90	1 + 2	111.8	13.6	88	Oligodendroglioma	2	Radiotherapy	Pressure ulcer	90	90	Stable
41	M	90	1	144.1	0	100	Glioblastoma	4	Radio-chemotherapy	–	100	90	Stable
42	F	90	Giant	178.0	15.5	91	Glioblastoma	4	Radio-chemotherapy	–	100	100	Stable
56	M	90	1	54.6	12.6	77	Oligodendroglioma	3	Radio-chemotherapy	–	100	90	Stable
27	M	90	Giant	150.5	27	82	Astrocytoma	2	MRI follow-up	Awakening delay, left arm paresis	90	100	Stable
45	M	90	3 + 4	43.0	5.4	88	Oligodendroglioma	2	Chemotherapy	–	100	100	Improvement
33	M	90	1 + 4	121.5	38.2	69	Astrocytoma	2	MRI follow-up	Transient hemiparesia	80	90	Decrease
32	M	90	Giant	211	26.3	88	Glioblastoma	4	Radio-chemotherapy	–	90	80	Decrease

Preoperative neuropsychological abnormalities were: language initiation weaknesses (n = 2), weakness of verbal working memory (n = 1), perturbations of long-term nonverbal memory (n = 1). All preoperative speech functional MRI confirmed a contralateral lateralization of language (left-sided speech areas even in left-handed patients).

Seventeen (85%) patients harbored de novo tumors (5 astrocytomas WHO grade II, 5 oligodendrogliomas WHO grade II, 2 astrocytomas WHO grade III, 1 oligodendroglioma WHO grade III, 4 glioblastomas WHO grade IV), and 3 (15%) presented with recurrences (2 astrocytoma WHO grade II, 1 oligodendroglioma WHO grade II). The tumor locations were depicted according to the Berger-Sanai classification, with a frontal predominance (15 located in zone 1) (Table 1)^16^.

The median follow-up was 29 months (IQR 20–34).

### Basic operative data, including IoMRI duration

All patients benefited from at least one IoMRI control. No incident was reported (in particular no magnetic incident and no integrated imagery platform dysfunction). The median duration of the first IoMRI control was 27 min (IQR 21–37). Fourteen (70%) patients benefited from a second IoMRI, which median duration was 20 min (IQR 19–29).

The median total operative time was 6 h 45 (min: 5 h 15; max: 8 h).

### Pre-, intra- and postoperative volumetric analyses and resection rates

The median preoperative volume was 64.5 cm^3^ (min: 9.9; max: 211 cm^3^). The median tumor volume after the 1st IoMRI control was 20.1 cm^3^ (min: 3.1; max: 84 cm^3^). Among the 14 patients who underwent a second IoMRI, the median tumor volume after the second IoMRI was 7 cm^3^ (min: 0; max: 38.2 cm^3^). The median final postoperative tumor volume was 4.3 cm^3^ (min: 0; max: 38.2 cm^3^). The median final postoperative EOR was 92% (min: 69%, max: 100%) (Table 1).

Among the seven patients harboring enhancing lesions after gadolinium infusion, no enhanced tumor residual was left.

### Residual tumor presence at the end of the surgery

In the majority of cases, the tumor residual (FLAIR hypersignal) was located in the posterior part of the insula (10 patients (50%)). The second frequent location of the tumor residue was fronto basal, medial to the insula (7 (35%) patients).

### Median hospital duration

The median hospital stay was 8 days (IQR 7–8 days).

### Early postoperative adverse events

Early postoperative clinical events (during hospital stay) were as follows: three transient left hemiparesis (lasted < 48 h), one prolonged left brachio-facial hemiparesis (with a KPS = 50 at discharge related to the ischemia of the anterior arm of the internal capsule. The motor deficit decreased in a few weeks and needed rehabilitation, the patient returned to work at 6 months postoperatively), two frontal syndromes (one apragmatism, one disinhibition), one decubitus ulcer related to the surgical positioning, one central diabetes insipidus.

### Postoperative KPS

The median postoperative KPS at discharge was 90 (IQR 80–90) (Table 1). At 3 months from surgery, the median KPS was 90 (IQR 90–100). All clinical events mentioned above resumed at 3 months. Among the 20 patients, 9 (45%) came back to work, between 15 days after the surgery to 14 months. Most of them needed to adapt their work rhythm due to fatigability.

### Neuropsychological outcomes

Regarding the postoperative neuropsychological reevaluation, among the three patients harboring preoperative symptoms, one of them presented with a stable exam and two improved their results concerning the episodic visual memory and had normal executive functions. Three other patients harbored new symptoms: the first one: visuoconstructive deficits and attention disorders (anaplastic oligodendroglioma WHO grade III, with postoperative radiotherapy performed before neuropsychological evaluation), the second one: executive and attention disorders (oligodendroglioma WHO grade II, received chemotherapy before neuropsychological evaluation), the third one: visuoconstructive and visual episodic memory disorders (glioblastoma, WHO grade IV, radiotherapy and chemotherapy performed before postoperative testing). These three postoperative deficits persisted at last examination (> 2 years after surgery).

**Figure 1:**
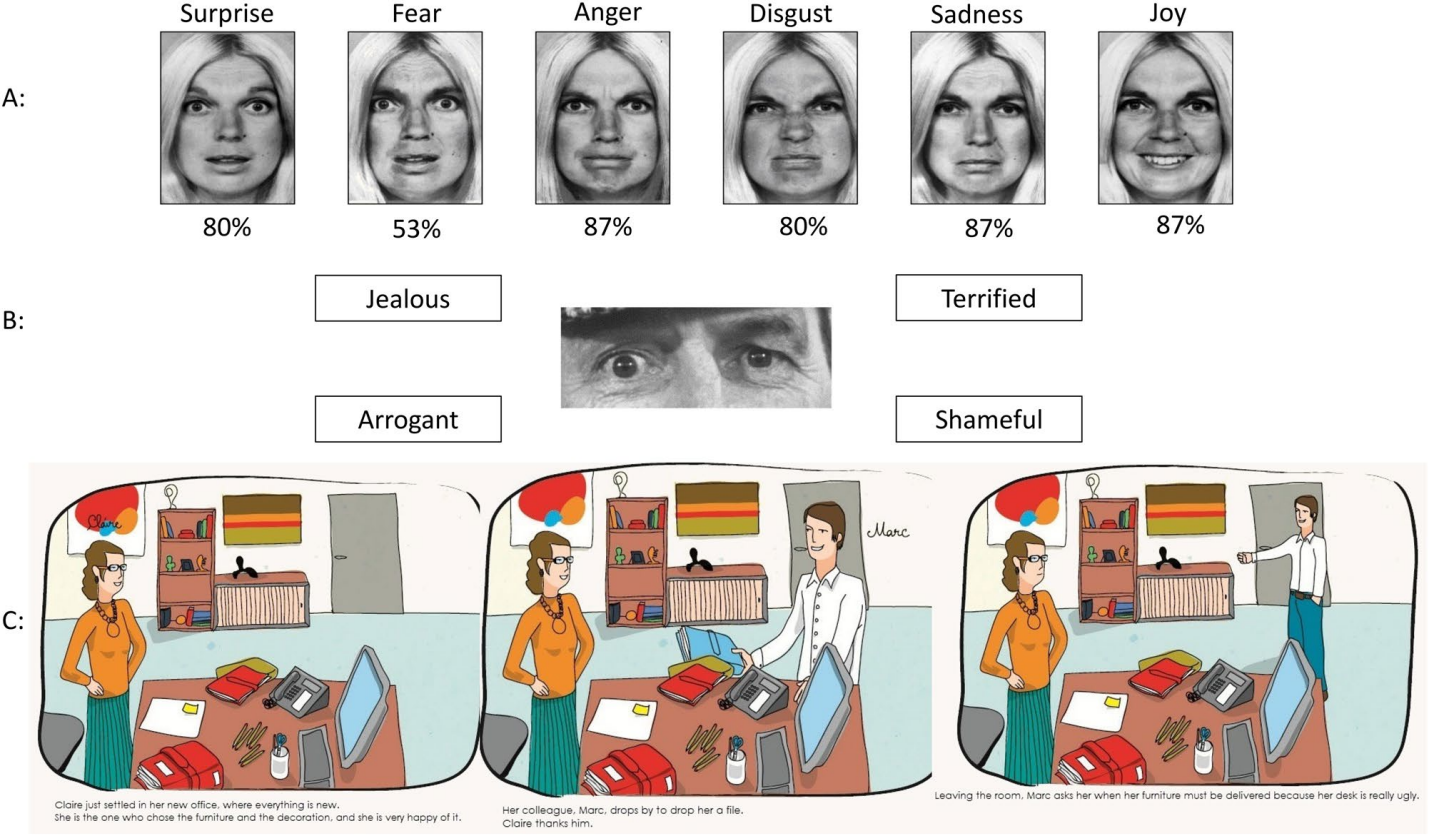
Illustrations from the battery of tests (from Ehrle et al. et Baron Cohen et al.). (**A**) In this task, a display shows faces based on Ekman faces of the same person expressing six basic emotions: anger, disgust, happiness, fear, surprise, and sadness. Faces are presented during a maximal duration of five seconds, followed by the presentation of emotion names. Participants have to decide which one of the six emotions best describe the face displayed. The percentage under each picture corresponds to the rate of successful recognition by the patients. (**B**) “Reading the mind in the eyes test” assessed perceptive-based mentalizing (Baron-Cohen et al.^31^). (**C**) “Theory of mind”: the examiner reads the text to the participant who can also read it simultaneously. The participant is then asked different questions assessing social cognition but also the understanding and retention of the story.

Concerning sociocognitive tasks, in the recognition of primary facial emotions task, a total of 80% (53–87%) of normal recognition was observed, with an important inter-individual variability (Fig. 1A). On the one hand, no patient showed a complete loss of recognition of all primary facial emotion, but on the other hand, no emotion was normally recognized by all patients. Recognition of the facial expression of fear harbored the most elevated rate of deficits (53% of correct recognition).

The “Reading the Mind in the Eye” test reported that 85% of patients were able to attribute complex mental affective and cognitive states from eye region photographs as accurately as the normative population (Fig. 1B).

Excluding understanding or memory difficulties, the “theory of mind” tasks reported that patients were able to successfully attribute mental states to others in false belief task of first order in 100% of cases and in second order in 92% of cases. Eventually, the faux-pas task showed the most elevated rate of deficits (success in 64% of cases). This was either due to the inability of some participant to detect the faux pas or failure on the question evaluating empathy, which required the participant to infer a character’s emotion (46 and 62% of success depending on the story).

### Further therapies after microsurgical resection

After surgery, depending on the tumor histology, ten patients underwent observational management, six received concomitant radio-chemotherapy and four had chemotherapy or radiotherapy alone.

## Discussion

In the present series, we report preliminary results in twenty patients harboring fronto-temporo-insular gliomas, all located in the non-dominant hemisphere and operated under general anesthesia using adjunct intraoperative MRI guidance. In our experience, the use of IoMRI helped us to maximize the EOR to more than 90% in a vast majority of cases. Moreover, the number of immediate postoperative complications was low (a total of four, of whom 3 were transient). With regards to the particular aspect of specific neuropsychological evaluation, this reported a high preservation rate of sociocognitive abilities even after large right FTI gliomas resection under general anesthesia and without any use of cortical or subcortical brain mapping. In the vast majority of cases, the residual tumor (as seen in FLAIR hypersignal) was located in the posterior part of the insula, while second frequent location was fronto basal, medial to the insula.

In our series, the median preoperative tumor volume was 64.5 cm^3^ (min: 9.9; max: 211). Compared with all gliomas undergoing IoMRI in our institution, FTI gliomas were significantly larger (64.5 cm^3^ vs. 26.3 cm^3^, p < 0.05)^17–19^.

Our volumes were higher than what was reported in the Berger's series (median volume of 48.5 cm^3^), but quite similar to Duffau’s series (median volume of 65 cm^3^)^8,16^. After the resection of the opercular part and the lobar part (frontal or temporal) of the tumor, we consistently observed an important "brainshift" with a loss of accuracy of the neuronavigation. At this point, we performed an IoMRI to update our navigation and to check the tumor remnant (strategic tumor remnant, staged volume surgery^19,20^). In almost all cases, a posterior and internal insula tumor residue was reported at this stage (at first IoMRI, median tumor residual was 20.1 cm^3^). Then, the dissection could be performed in a safe manner aiming at a maximal resection rate. During the second IoMRI check, the median tumor residual dropped to 7 cm^3^. No significant brainshift was reported between the first and second IoMRI. As illustrated in Fig. 3, consecutive MRI controls helped to reduce the tumor residue with a postoperative median residue of 4.3 cm^3^. Our final resection rate was of 92%, which is higher than in the previous publications concerning insular gliomas^7,21^. Berger et al. reported an 85% EOR in a series of 218 patients harboring FTI gliomas operated on without intraoperative imaging^16^. Ius et al. reported an 80% EOR using intraoperative cortical and subcortical electric stimulation associated to neurophysiological monitoring^21^. In Ius series, surgeries were also performed under general anesthesia in the non-dominant hemisphere. In this context, our present data suggests that FTI gliomas in the non-dominant hemisphere could be operated on using IoMRI under general anesthesia without electrophysiological mapping, reaching high EOR with favorable functional outcomes^18^.

In our experience, the use of IoMRI was particularly beneficial for low-grade gliomas, to help delineate the tumor boundaries^20^. Clinical benefit of aggressive surgical resection is dependent on the visibility of the tumor boundary, involvement of perforators (such as long insular artery and lenticulostriate artery) and extent of the tumor invasion adjacent to the eloquent area determined by Berger–Sanai classification system. The difference of invasive patterns between frontal and temporal side should have a strong impact on neurophysiological function and resection strategy^15^. As a consequence, we adapted our surgical strategy to the tumor invasiveness. In case of a predominant frontal tumor contingency (type 1 Berger-Sanai), we performed a frontal trans opercular approach, with special care to the long insular arteries. Our posterior border of dissection was the pars opercularis in order to avoid damaging the ventral motor cortex. Facing a predominant temporal tumor (type 4 Berger Sanai), we dissected through the temporal operculum, with less restriction than in the frontal posterior location.

Berger et al. reported higher EOR for gliomas located in the anterior part of the insula (zones 1 and 4 according to Berger-Sanai). Our results are in line with this finding, as we report higher EOR in our series because of frontal predominant tumor location. As expected, most of our residual tumors were located in the posterior and internal part of the insula (Table 1).

It has been reported that low-grade gliomas of the insular region benefit from an EOR > 90%, which further significantly increased 5-year survival rates^7^. The same was true for high-grade gliomas^7^. Thus, the use of IoMRI may have a favorable impact on patient survival. In our series 60% of patients harbored an EOR > 90%. Performing asleep surgery with IoMRI guidance for right FTI glioma, we provide maximal EOR without additional functional impairment, and then set the patient in optimal condition for receiving adjuvant therapies or following MRI monitoring.

Some advocate for preoperative tumor biopsy to get pathological/genetic diagnoses which might provide prediction for sensitivity of postoperative adjuvant therapy^15^. For instance, oligodendroglioma is known to be a dormant tumor and sensitive to procarbazine-CCNU-vincristine chemotherapy (PCV). The glioma surgeon should consider that all patients with oligodendroglioma are able to go back to ordinal life, even if extent of resection is less aggressive.

Nineteen of the 20 operated patients had a KPS ≥ 80 at discharge. In this respect, in this small series, our results are comparable to previously published data of a recent meta-analysis, reporting a range of postoperative neurological deficits after glioma resection under intraoperative stimulation mapping, between 3.4 and 8.2%^22^.

However, three patients harbored transient left hemiparesis and a fourth patient had persistent brachio-facial paresis. In the above-mentioned cases, early postoperative MRI reported areas of recent ischemia at the edge of the operative cavity. One of the major risks of insular tumors dissection is the involvement of lenticulostriate arteries. In addition to careful surgical dissection, preoperative recognition of the positioning of lenticulostriate perforating branches is essential in order to avoid the unexpected occurrence of postoperative ischemic stroke. Some authors recommended additional digitized cerebral arteriography with fusion of MRI volumetric data^23^. Awake surgery does not prevent from such vascular injury and particular care should be given in this context. However, motor evoked potentials (MEP) could be of interest to assess in a real time manner the occurrence of a symptomatic spasm or vascular injury. Concerning the MCA perforator lesions, we avoid dissecting to deeply in the fronto basal area posteriorly to the MCA. In the opercular region, the use of ultrasonic dissector could be of interest to reduce the risk of long perforator arteries lesions.

One patient experienced a transient postoperative diabetes insipidus. We explain this metabolic disturbance by a deep and medial dissection near the hypothalamic region which could have hampered hormone homeostasis.

In our series, no phasic disorder was reported. The use of functional MRI proved reliable to identify the dominant hemisphere and allowed to perform such tumor resection in asleep condition when the tumor was located in the non-dominant hemisphere^24,25^. Mahvash et al. reported correct dominant hemisphere identification in 90% of cases; using an imaging protocol lasting less than 10 min^25^. However, the activation zones described have no localization value, which is why we did not use this technique alone in dominant hemisphere surgery^18,26^. Moreover, for FTI gliomas located in the dominant hemisphere, we always perform awaked cortical and subcortical mapping^7,8^.

Further evaluation (at a time point of more than 3 months after surgery) of functional outcomes was difficult, due to the different postoperative patient care. Some patients received adjuvant therapies such as combined radio-chemotherapy whereas other patients underwent MRI follow-up depending on the tumor grade and the EOR. Larger distinct series of low-grade and high-grade gliomas could be analyzed to better assess respective long-term outcomes.

The pre-and postoperative neuropsychological testing (performed in the first three months after surgery) helped to detect inconspicuous neurocognitive deficits. According to our results, the surgical removal of such FTI gliomas could improve the neuropsychological evaluation at three months (improvement of language initiation and verbal working memory). Recent series, such as Motomura et al. and Satoer et al. reported the same postoperative neuropsychological improvement, especially regarding visual memory and delayed recall ^27,28^.

Nevertheless, three patients harbored new postoperative symptoms such as visuoconstructive troubles, attention disorders and visual episodic memory impairment. These deficits were persistent at last follow-up. One could explain, at least in part, these alterations by the postoperative treatments such as radiotherapy and chemotherapy performed before the postoperative neuropsychological testing. Wu et al. studied the impact of insular gliomas on neurocognitive functioning in comparison with other glioma locations^29^. These authors reported that patients harboring insular glioma had a more severe impairment of preoperative naming tasks in comparison with other glioma locations. In the postoperative period, Wu et al. described increased difficulties for these same patients in learning and memorizing tasks. According to Wu’s series, FTI gliomas in the non-dominant hemisphere harbored postoperatively greater decline in the domain of visuoconstruction. Therefore, on a series of 42 insular gliomas, Duffau et al. noted an early postoperative worsening of the neuropsychological assessment, which recovered before 3 months^13^. This recovery was explained by the author by the neural plasticity potential of the insula as well as the compensation phenomena based on the ipsilateral opercular structures and the contralateral insula^30^. However, this series also included gliomas of the dominant hemisphere with increased risk of impairment of language functions. Some other authors, like Duffau et al. advocated for cortical and subcortical stimulation in the right hemisphere to avoid “mentalizing” impairment (difficulty for patients to recognize facial emotions)^13,31^. According to us, the sociocognitive functions cannot be defined in a localizationist theory. These functions belong to complex neural network, called hodotopy. It seems misleading to identify all this complex network with direct electrical stimulation. In our experience, testing such subtle cognitive functions is demanding, even for the patient and the medical staff. Cortico-subcortical stimulation for such purpose can be long-lasting, especially in case of large/giant glioma according to Berger–Sanai classification (e.g. Fig. 3: case illustration).

**Figure 2:**
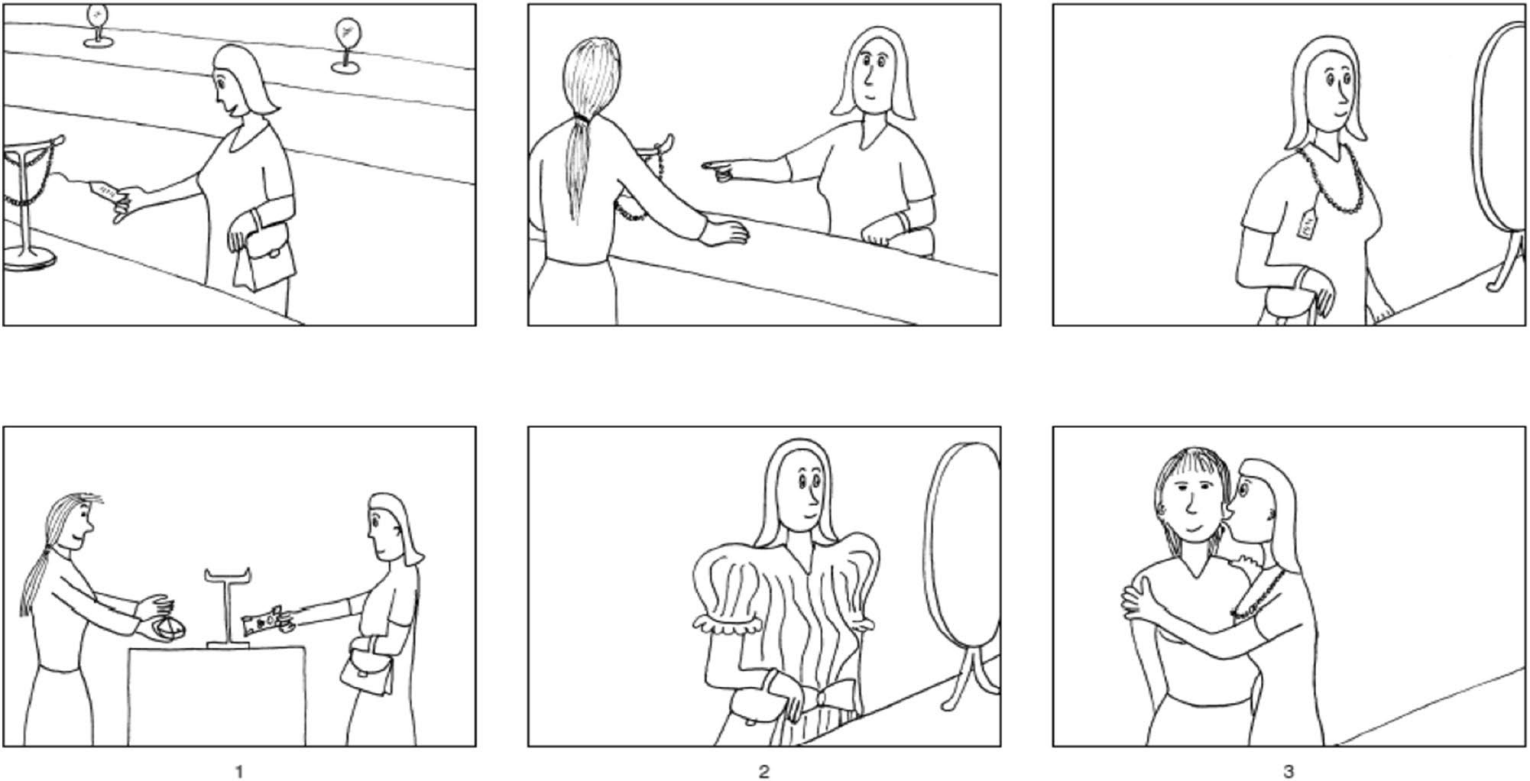
V comics: this test was used to assess inference-based aspects of mentalizing. In this task, participants are required to infer a character's intention, or to make inferences based on physical causality. (From Ehrle et al. et Baron Cohen et al.)

To assess the sociocognitive function pre- and postoperatively, we used advanced validated tests. The French school of neuropsychologist has a long tradition in evaluating social behavior and its neurological implications^14,32^. Visuo-constructive apraxia was evaluated by the recognition of the six primary facial emotions (Fig. 1A). Complex mental affective and cognitive states were evaluated with the eye region photographs^33^ (Fig. 1B). Then the “theory of mind” and empathy were assessed thanks to false belief tasks of first and second order and faux-pas tasks using six illustrated stories (Fig. 1C). Finally, the V-Comics was used to assess participants’ ability to infer a character's intention, using strip cartoons (Fig. 2). This kind of specific and complex testing seems hard to reproduce intraoperatively. In our experience, after dedicated sociocognitive testing, no postoperative significant impairment was reported in a majority of patients. Normal face recognition was observed in 80% of cases. Fear was the least well-identified emotion (53%). Recent researches, including functional MRI, reported that the insular cortex plays a main role in the identification and reaction to fear^34,35^. Complex mental and cognitive states accurately was attributed in 85% of cases. The only difficulty was the inability of some patients to correctly evaluate empathy. Accumulating evidence highlights a crucial role of the insular cortex in feelings especially empathy^36^. It is worthy to mention that some patients received chemotherapy or radiotherapy or both before the postoperative neuropsychological testing, as the series also included high-grade gliomas (e.g., 4 glioblastomas). According to our present results and in this small preliminary series, IoMRI guided surgery under general anesthesia for FTI glioma resection in the non-dominant hemisphere might represent a consistent alternative to awake surgery, especially for large tumors.

Regarding the patient’s socio-professional evolution, 45% came back to their previous work. The reasons why some patients were not able to return to work were neither neurologic deficit nor neuropsychological disablement. These patients faced some difficulties with their employer to accept their disease or had to deal with complex familial context. Few patients harbored residual seizures. In some cases, adjuvant therapies such a radiation therapy or chemotherapy hampered the patient autonomy. Taking into account the type of lesion and the potential complication related to the surgical excision, these functional outcomes were encouraging.

This research included only 20 FTI glioma cases. Our series embraced different grades of glioma. As a matter of consequence, it implies a certain heterogeneity in the patient medical management, especially regarding the adjuvant therapies (radiotherapy and/or chemotherapy). Most patients harboring WHO grade III or IV glioma received radiotherapy before the postoperative neuropsychological examination. It could interfere with their testing performance. Due to the series heterogeneity, we did not report the progression-free survival and the overall survival that would have little value in this context. Moreover, we used “Observer reported outcomes” (ORO) to evaluate the KPS. To better assess complaints and patient quality of life, we could also have used “Patient reported outcomes” (PRO)^37^. Repeated neuropsychological testing could be of interest as previous series reported neurocognitive improvement even after three months after surgery^38^.

**Figure 3:**
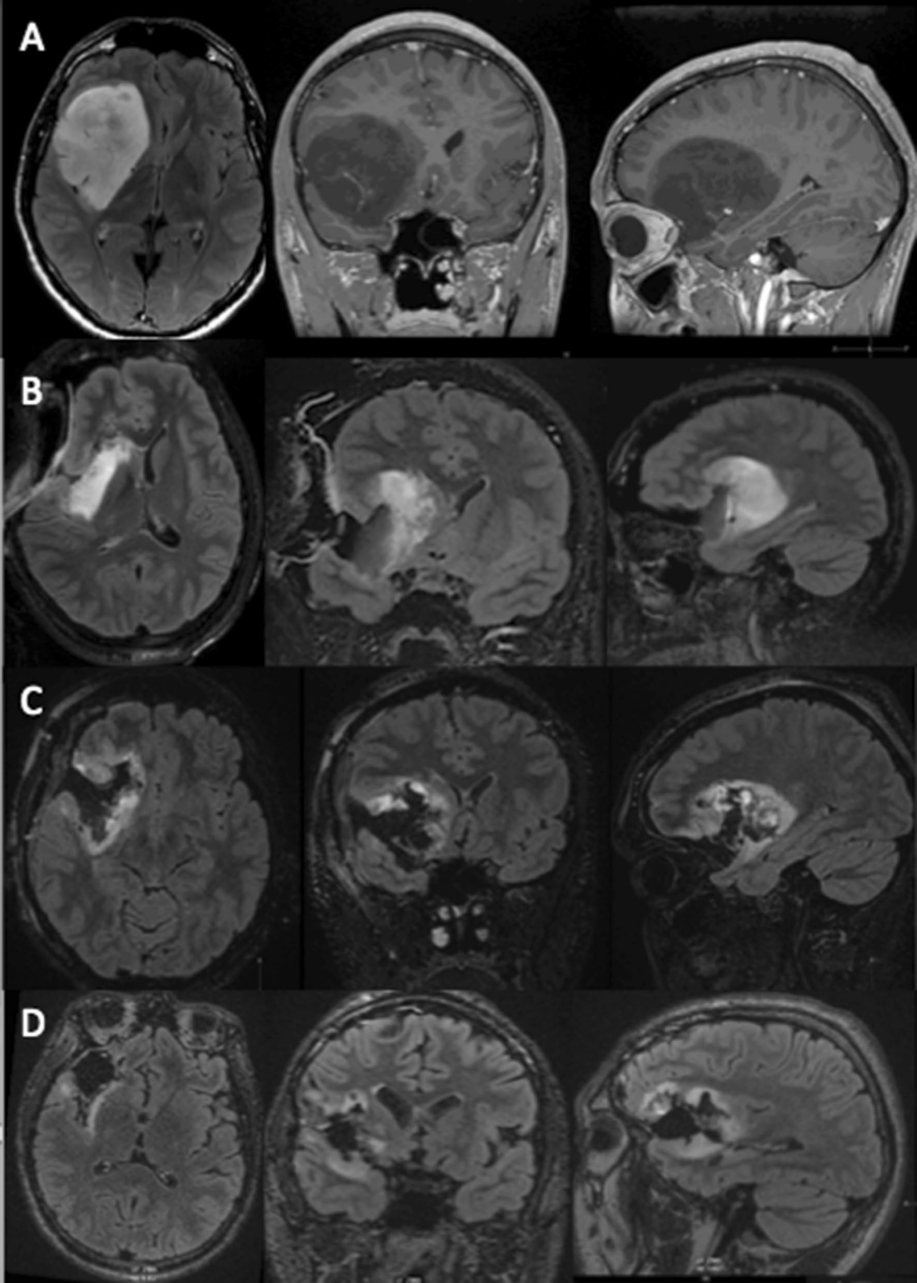
Staged surgical approach for a right fronto-temporo-insular glioma. (**A**) Preoperative MRI depicting a hypo signal T1 lesion, with sharp contours. The preoperative volume was of 112 cm^3^. (**B**) 1st IoMRI reporting a tumor remnant at the inner and posterior part of the insula and in the frontal lobe of 38 cm^3^. Additional resection was performed. (**C**) 2nd IoMRI, the tumor residue was of 11 cm^3^. Due to the presence of the internal capsule at the superior part of the lesion the end of surgery was decided. (**D**) Postoperative control at 3 months, with less perioperative signal abnormalities the residue was reassessed, with a volume of 6 cm^3^, corresponding to an EOR of 95% (using IntelliSpace Portal, Philips, module “tumor tracking.” version 9.0 (www.philips.fr).

This publication does not provide a control group and this for several reasons. As the use of IoMRI has been previously reported as significantly improving the extent of resection for glioma^12,39^, we did not perform FTI tumor resection without such an adjunct. In our university center, every single low-grade glioma is operated on with IoMRI guidance since 2014. For this reason, a control group using navigation without IoMRI was not an option for us. Regarding intraoperative awake mapping, this surgical technique has been widely spread based on uncontrolled studies^40,41^. We aimed to compare our results to these reported studies, evaluating awake mapping without control group. Recently, Motomura et al. reported the same kind of neurocognitive preservation using functional mapping in glioma surgery, without a control group^27^. Moreover, such fine awake intraoperative neurocognitive testing seems questionable according to our experience. Complex cognitive tasks, such as social cognition, also known as mentalizing, could not be tested directly with an electric probe. Latest research on the subject put forward quantum mechanics as a way to conceptualize consciousness^42–44^. Indeed, sociocognitive testing needs a high level of concentration for a significant time. Unlike language, such complex functions could not be assessed in a few seconds and in a repetitive manner. Finally, we already experienced the combine use of awake mapping and IoMRI. When performing resection of large FTI gliomas (e.g. Fig. 3) in awake conditions with the concomitant use of IoMRI, patients were sometimes not able to pursue the neuropsychological testing after the 1st IoMRI due to fatigue and stress. We were then forced to continue the surgery under general anesthesia.

## Conclusion

Our series is small, but raises the question whether FTI gliomas of the non-dominant hemisphere could be operated in asleep condition, under general anesthesia and using the adjunct of IoMRI, while using no cortical or subcortical mapping.

In sum, the use of IoMRI helped to reach high resection rates, of more than 90%, in a majority of cases. The vast majority of tumor remnants were located in the posterior insular part. The reported neurological morbidity was minor and frequently transient. The specific neuropsychological evaluation reported a high preservation of sociocognitive abilities even after large right frontal resection. Solely empathy seemed to be impaired in some patients.

## Materials and methods

All methods were carried out in accordance with the applicable guidelines (STROBE statement), in compliance with the Declaration of Helsinki. Our institutional ethical committee approved this study (Comité d’éthique pour la recherche de l’Université de Lille). The written informed consent was obtained from all the patients in the study. Informed consent for publication of identifying information/images in an online open-access publication has been obtained.

### Patients

We prospectively collected clinical (including neuropsychological evaluation) and imaging data for all patients operated on for a right FTI glioma (non-dominant hemisphere in all patients) under general anesthesia and using IoMRI adjunct. In fact, our hospital (Lille University Hospital, Lille, France) was the first in France to benefit from the installation of IoMRI as early as July 2014.

Patients benefited from microsurgical resection from July 2014 to December 2018, at our institution. All were examined by a board-certified neurosurgeon (NR, HAL), to assess the Karnofsky Performance Score (KPS) before and after surgery. The patients benefited from a pre- and a postoperative neuropsychological evaluation assessing patient complaints (cognitive, behavioral or mood) and reported the impact on daily life. In particular, the neuropsychological assessments detailed: overall cognitive efficiency (Montreal Cognitive Assessment, MoCa), the working memory, verbal and visual memory, learning, executive tasks, attention, language (speaking and listening), visuoconstructional and gnosic functions, and reasoning. The same neuropsychologist (OS, second author) reassessed the patients at least once, at 3 months after surgery. Concerning sociocognitive assessment, the “three tasks” issued from the “Batterie de Cognition Sociale,” a standard and well-recognized French battery of tests, were used^14,32^ (Fig. 1, Annex 1). This test evaluated the recognition of six primary facial emotions, the attribution of complex mental affective and cognitive states from eye region photographs^33^ and “theory of mind” and empathy thanks to false belief tasks of first and second order and faux-pas tasks using six illustrated stories. Finally, the V-Comics was used to assess participants’ ability to infer a character's intention, using strip cartoons (Fig. 2).

Preoperatively, all patients had a 3D MRI used for navigation and a functional MRI, displaying the blood-oxygen level dependent (BOLD) activation of language areas to confirm the contralateral lateralization of language.

In sum, all patients had right FTI gliomas and localization of language as depicted by fMRI on the left hemisphere, independently of being right- or left-handed.

In case of left-sided glioma, awake surgery was performed and the patient was not included in this study.

The postoperative clinical outcomes have been reported using the same KPS score and specifying the occurrence of a new or increased neurological deficit.

### Surgical workflow

The way of using our operative room platform, including IoMRI, was previously reported by Leroy et al. and Reyns et al.^18,20^.

Various surgical approaches were performed to resect these 20 fronto-temporo-insular gliomas. The choice was based on the main contingent of the tumor. If the main tumor contingent was frontal, we opted for a frontal transopercular approach. If it was temporal, a superior temporal resection was performed. If necessary, both frontal and temporal transopercular approaches could be performed. Some authors reported that dissection of at least a portion of the Sylvian fissure may be necessary to expose the tumor boundaries and enlarge the surgical window without sacrificing Sylvian bridging veins^45,46^. This step makes possible to locate the upper and lower periinsular sulcus that delimited the upper and lower dissection planes. Locating these periinsular sulci can further comfort the surgeon in his spatial orientation, especially in the case of a shift of the neuronavigation linked to a "brainshift". The IoMRI helps to overcome this difficulty by updating neuronavigation, once coregistered with the preoperative MRI used for the initial surgical step. We therefore did not expose the periinsular sulci systematically. No brain retractor was used. Subpial tumor dissection was performed using a thin bipolar and a Brachman suction (size 15 or 20). Then we used the ultrasonic dissector at a low power to complete the resection.

One or two IoMRI was performed, especially to access the posterior part of the insula (near the internal capsule and the basal ganglia). In this surgically difficult access and functional area, we voluntarily left a remnant before reassessing the neuronavigation data, based on IoMRI. This way of dissection relying on a step by step tumoral removal (staged volume surgery) was called “strategic tumor remnant”^20^. It allowed us to work with an accurate neuronavigation and increase the EOR.

### Volumetric analyses

Two independent observers (one senior neuroradiologist, QV, one senior neurosurgeon, HAL) measured all preoperative, intraoperative and postoperative glioma volumes. We used the software IntelliSpace Portal, Philips, module “tumor tracking.”, version 9.0 (philips.fr). The lesions were contoured on T2/FLAIR sequences. After segmentation, volumes were calculated in cubic centimeters. If a discrepancy > 10% was reported between the two observers, we compared both our segmentation to achieve a consensus volume. We also reported the extent of resection (EOR) in percentages. The potential postoperative tumor remnant was defined on early postoperative MRI, performed in less than 48 h after surgery.

### Statistical analyses

The institutional biostatistics department performed the statistical analyses. Qualitative parameters were described as frequency and percentages. The quantitative parameters were described as median and associated interquartile range (IQR). Comparisons between patients were performed using the Mann–Whitney U test for quantitative variables and using the chi-square test for qualitative variables*.* Statistical testing was done at the two-tailed α level of 0.05. The data were analyzed using the SAS software (SAS Institute Version 9.4) (www.sas.com).

## Supplementary Information

Supplementary Information.
