# Supplementary material for: Microsurgical resection of fronto-temporo-insular gliomas in the non-dominant hemisphere, under general anesthesia using adjunct intraoperative MRI and no cortical and subcortical mapping: a series of 20 consecutive patients
Source: Sci Rep. 2021 Mar 26;11:6994. doi: 10.1038/s41598-021-86165-7 (PMC7997967; doi:10.1038/s41598-021-86165-7)
Supplement: Supplementary file 1 — Supplementary Information. [file 41598_2021_86165_MOESM1_ESM.doc]

Microsurgical resection of fronto-temporo-insular gliomas in the non-dominant hemisphere, under general anesthesia using adjunct intraoperative MRI and no cortical and subcortical mapping: a series of 20 consecutive patients

Authors:

Henri-Arthur Leroy, MD^1, 2^, Ondine Strachowksi^1^, Constantin Tuleasca^1,3^, Quentin Vannod-Michel^4^, Emilie Le Rhun, MD^1^, Benoit Derre^1^, MSc, Jean-Paul Lejeune, MD, PhD^1^, Nicolas Reyns, MD, PhD^1, 2^

Affiliation:

^1^CHU Lille, Department of Neurosurgery and Neuro-oncology, F-59000 Lille, France

^2^Univ. Lille, Inserm, CHU Lille, U1189 - ONCO-THAI - Image Assisted Laser Therapy for Oncology, F-59000 Lille, France

^3^Department of Clinical Neurosciences, Neurosurgery Service and Gamma Knife Center, Lausanne University Hospital (CHUV) and University of Lausanne (Unil), Faculty of Biology and Medicine (FBM)

^4^CHU Lille, Department of Radiology, F-59000 Lille, France

**Annex 1**

The first three tasks are part of the BCS (Ehrlé and al., 2011), a French computer battery designed to assess emotional and sociocognitive abilities.

**Recognition task of primary facial emotions**. In this task, a display shows faces based on Ekman faces (Ekman and Friesen, 1976) of the same person expressing six basic emotions: anger, disgust, happiness, fear, surprise, and sadness. It contains 60 stimuli, 10 for each emotion, corresponding to different expressive intensities. Faces are presented during a maximal duration of 5 seconds, followed by the presentation of emotion names. Participants have to decide which one of the 6 emotions best describes the face displayed. They are asked to answer orally as fast as possible as their response time is measured. The examiner records participants’ response. Training for each emotion with feedback precedes the test. Stimuli are then displayed in a pseudo-random order, with 2 successive stimuli maximum belonging to the same emotion.

**Reading the Mind in the Eye Test (RMET)**. This test was used to assess perceptive-based mentalizing. A French adaptation of the revised version of the Reading the Mind in the Eyes Test (Baron-Cohen et al., 2001) was administered. In this task, participants have to identify which of 4 words describing complex mental affective and cognitive states best matches the intention expressed by a photograph of the eye region of a person. A definition of each word with a synonym and an example is available to the participant if needed. A total of 33 stimuli are presented. Response and response time are measured. An example with feedback precedes the test.

**Theory of mind**. Three different tasks assessing attribution tasks of mental states to others are used: false belief tasks of first and second order and faux-pas task, using 6 stories (2 of each) illustrated by drawings displayed on a screen. The examiner reads the text to the participant who can also read it simultaneously. The participant is then asked different questions assessing social cognition but also the understanding and retention of the story.

**V-Comics**. This test was used to assess inference-based aspects of mentalizing. In this task, participants are required to infer a character’s intention, or to make inferences based on physical causality. The Brunet’s modified version of the comic strip task (Brunet et al., 2000) consisting in strip cartoons was used. Each strip cartoon consists of three pictures in the upper half of the page. Three other pictures are presented in the lower half showing three possible endings. Participants have to select the picture from the three alternatives that shows the more likely ending of the plot. There are 34 strip cartoons divided into three conditions according to the type of inference required for selection. Intention mentalizing (14 stimuli), which requires participants to select the ending that shows the most probable next action of the person. Physical reasoning which requires participants to select the story ending that best follows physical rules, with a condition including a character (10) and another condition with no character (10). A training session with 5 items (including intention mentalizing and physical reasoning with and without character) is performed before the test.
